# Do changes in frailty, physical functioning, and cognitive functioning predict mortality in old age? Results from the Longitudinal Aging Study Amsterdam

**DOI:** 10.1186/s12877-022-02876-0

**Published:** 2022-03-12

**Authors:** Sasmita Kusumastuti, Emiel O. Hoogendijk, Thomas A. Gerds, Rikke Lund, Erik L. Mortensen, Martijn Huisman, Rudi G. J. Westendorp

**Affiliations:** 1 Section of Epidemiology, Department of Public Health, University of Copenhagen, Copenhagen, Denmark; 2 Center for Healthy Aging, University of Copenhagen, Copenhagen, Denmark; 3 Department of Epidemiology and Data Science, Amsterdam Public Health Research Institute, Amsterdam UMC – location VU University Medical Center, P.O. Box 7057, 1007 MB Amsterdam, Netherlands; 4 Section of Biostatistics, Department of Public Health, University of Copenhagen, Copenhagen, Denmark; 5 Section of Social Medicine, Department of Public Health, University of Copenhagen, Copenhagen, Denmark; 6 Department of Public Health, Section of Environmental Health, University of Copenhagen, Copenhagen, Denmark

**Keywords:** Functional decline, Health indicators, Prognosis, Survival analysis, Frailty

## Abstract

**Background:**

The ability to accurately predict survival in older adults is crucial as it guides clinical decision making. The added value of using various health indicators as well as changes in these indicators for predicting mortality remains unclear. The aim of this study was to investigate whether changes in health indicators such as frailty and physical performance improve mortality predictions in old age.

**Methods:**

This is a population based prospective cohort study on 995 community-dwelling people aged 68–92 years from the Longitudinal Aging Study Amsterdam. Two measurements at a three-year interval (1995/1996 and 1998/1999) were available for the frailty index, frailty phenotype, grip strength, walking speed, and Mini-Mental State Examination (MMSE). Cox regression was used to analyze mortality risks associated with the current health status and changes in health, with mortality data up to 2017. The extent to which these health indicators improved mortality predictions compared to models with age and sex only was assessed by the area under the receiver operating characteristic curve (AUC).

**Results:**

The AUC of age and sex for five-year mortality was 72.8% (95% CI 69.0 – 76.5) and was the lowest in the oldest old (age > 80.5 years). The added AUC of the current status of health indicators ranged from 0.7 to 3.3%. The added AUC of the three-year change was lower, ranging from -0.0 to 1.1%, whereas the added AUC of three-year change and current status combined was similar to current status alone, ranging from 0.6 to 3.2%. Across age, the added AUC of current status was highest in the oldest old, however there was no such pattern using three-year change. Overall, the frailty index appeared to improve mortality predictions the most, followed by the frailty phenotype, MMSE, grip strength, and walking speed.

**Conclusions:**

Current health status improved mortality predictions better than changes in health. Its contribution was highest in the oldest old, but the added value to models with age and sex only was limited.

**Supplementary Information:**

The online version contains supplementary material available at 10.1186/s12877-022-02876-0.

## Introduction

The ability to accurately predict survival is crucial as it guides decision making on the timing, character, and intensity of medical interventions. In old age, the need for prognostication becomes even more pertinent as older individuals have accumulated bodily damage due to the aging process resulting in complex manifestations of several chronic diseases and subsiding resilience to potential complications [1]. Consequently, over the past decades there has been a rise in studies developing, comparing, and upgrading stand-alone or composite arrays of health indicators in search of tools that provide the highest accuracy to predict survival in old age [2, 3]. These prognostic tools encompass various health indicators ranging from genetic variation, tests of physiological and cognitive function, presence of diseases, exposure to environmental factors, and self-reported measures of health [4]. The general conclusion of the studies performed so far affirmed the advantage of using these instruments for prognostication and studies recommended their use to identify older adults at risk in daily clinical practice [5, 6].

Despite the general consensus on using current prognostic tools, the added value of using various health indicators for predicting mortality in every day medical services remains unclear. Recent findings have shown that accuracy of the tools in predicting mortality decreases with increasing age [7, 8]. In addition to this diminishing accuracy, very few studies have distinguished between demographic variables (e.g. age, sex, etc.) and health indicators when assessing accuracy [9]. It is important to make this distinction because age is the most significant risk indicator of functional decline, disease, disability, and mortality. Here the main question is to what extent we gain more accuracy in predicting mortality when these prognostic tools are applied, and to what costs [10]. Most studies so far have focused on using the health status at one point in time to predict outcomes in time thereafter. However, several studies have shown that changes in health status may have substantial effects on functioning and can serve as warning signs in identifying older adults at risk of decline and death [11–13]. For example, some recent studies demonstrated the added value of measuring changes in frailty for predicting mortality [14, 15].

The aim of the current study was to investigate the added value of change in health indicators to all-cause mortality predictions in old age based on demographic variables (age and sex) only. Specifically, the health indicators of interest are in the domains of physical and cognitive functioning since age-related decline in these indicators constitute some of the most severe problems (and threats to independent functioning) associated with growing older.

We have based our analyses on a prospective cohort study sample from the Longitudinal Aging Study Amsterdam (LASA) and evaluated the performance of change in the most commonly used functioning measures capturing frailty (the frailty phenotype and the frailty index), upper and lower body strength (grip strength and walking speed), and cognition (Mini-mental State Examination (MMSE)) to accurately predict mortality.

## Methods

### Study population

This study was based on the LASA cohort, a nationally representative cohort from the Netherlands of individuals aged between 55 to 85 years at baseline [16]. Respondents were initially selected from municipality registers of three regions: the western part of the Netherlands (in and around Amsterdam), the northeast (in and around Zwolle), and the south (in and around Oss). The primary aim of LASA was to study the determinants and consequences of aging with a focus on physical, cognitive, emotional, and social functioning [17]. Data were collected in measurement ‘waves’ since 1992/1993, in which participants were visited approximately every three years at their own home by trained interviewers. Data collection consisted of one visit involving a main interview (including basic physical performance tests, such as gait speed), one visit involving a medical interview with additional performance and cognitive tests among other things, and a self-reported questionnaire. More details on the sampling and measurements of LASA can be found elsewhere [16, 17]. For the current study, data from the various interview types were combined. The sample consisted of older individuals who participated at the follow-up measurements in 1995/1996 and 1998/1999. These consecutive waves were selected because we were interested in change of health indicators that were not available at all earlier or later waves. Furthermore, we chose a 3-year follow-up period to measure change in health indicators since this short time period is a better representation of the clinical situation. Additionally, attrition between these waves was mainly due to mortality [16]. Herein we included participants aged 65 years and older (in 1995/1996) who participated in both main and medical interviews with complete information on demographic variables, health indicators at both measurement waves (1995/1996 and 1998/1999), and survival outcome resulting in a study sample of 995 participants (see Supplementary Fig. S1). Since there were few older adults with missing information on some of the health indicators (*N* = 119), we decided not to perform multiple imputations. These 119 people with missing data were slightly older than the included sample, but the difference was not statistically significant. The LASA study received approval by the medical ethics committee of the VU University medical center, Amsterdam (file number 92/138). All participants signed a written informed consent.

### Health indicators

The health indicators included validated, well-known frailty instruments, i.e. the frailty phenotype and the frailty index; grip strength and walking speed as measures of upper and lower extremities functioning; and the Mini-Mental State Examination (MMSE) as a measure of cognitive functioning. The frailty phenotype was based on the presence of Fried’s frailty criteria: weight loss, weak grip strength, exhaustion, slow gait speed, and low physical activity with scores ranging from 0 (no frailty criteria) to 5 (all frailty criteria present) [18]. The frailty phenotype used in this study was slightly adapted compared to original instrument, and has been previously operationalized and validated in LASA [19–22]. In particular, the slow gait speed and low physical activity items of the frailty phenotype were adapted, following the lowest quintile approach instead of using the original cut-points of Fried and colleagues [19–22]. The frailty index has also been operationalized and validated in LASA [23], resulting in a 32-item index measuring the accumulation of symptoms, diseases, disabilities, or any other age-related health deficits. All deficits were given a score of 0 (absence of deficit) or 1 (presence of deficit) and the frailty index was calculated by dividing the sum of the health deficit scores by the total number of health deficits resulting in a range from 0 (no deficits present) to 1 (all deficits present) [24]. More details on the items included in the frailty index are provided in Supplementary Table S2. Grip strength was measured using a dynamometer and recorded to the nearest 1 kg, calculated using the sum of the highest values of the two hands divided by two. Walking speed was measured by recording the number of seconds it took participants to walk 3 m, turn around, and then walk back 3 m as quickly as they can. The MMSE consisted of 20 items examining orientation in time and place, words registration and recall, attention and calculation, language, and visual construction with scores ranging from 0 to 30 [25]. A higher frailty phenotype score, a higher frailty index score, slower walking speed, lower grip strength, and lower MMSE scores indicated worsening health. For each health indicator, we used health indicator scores in 1998 as the “current status” and “three-year change” was defined as the difference in health indicators scores by subtracting the measurement in 1998 minus the measurement in 1995. See Supplementary Fig. S3 for study design scheme.

### Statistical methods

We described median, interquartile range (IQR), minimal, and maximal values for continuous variables and counts for categorical variables. Time zero of all survival analyses was set at the date of the second follow-up measurement in 1998/1999. Participants were followed through direct linkage with registers of the participants’ municipalities of residence until death or March 1^st^ 2017, whichever came first. The Kaplan–Meier method was used to describe overall survival among groups based on age and sex. We also plotted mortality risks given a person’s current status and three-year change.

The reference model was obtained with Cox regression in the training set using additive effects of age and sex. The effect of age was modeled using restricted cubic splines with three knots [26]. For each health indicator, three Cox regression models were fitted by adding to the age-sex benchmark model the current status, the three year change, and both the current status and the change, respectively. The discrimination ability of five- and ten-year mortality predictions of the Cox regression models was assessed by area under the receiver-operating-characteristic curves (AUC) for right censored data in the test [27]. AUC indicated the probability that a participant who died within *t-*years was assigned a higher predictive risk of mortality when compared to a participant who survived for longer than *t*-years. The improved predictions of a health indicator was assessed by differences in AUC compared to the reference model and tested by a Delong-Delong type test [28]. P-values indicate the significance of the difference between a model containing age and sex plus a health indicator and the reference model that is age and sex only given the null hypothesis that the health indicator has no added discrimination ability. We also performed stratified analysis based on tertiles of age, sex, and number of chronic diseases (major chronic diseases, including: heart disease, peripheral arterial disease, stroke, diabetes, chronic lung disease, arthritis, cancer). All statistical analyses were performed using R version 4.0.2 [29].

## Results

Table 1 presents the characteristics of the study sample, which consisted of 995 participants aged 68.0 through 91.6 years at study baseline with an overall average of 77.4 ± 6.2 (SD) years and the 25^th^, 50^th^, and 75^th^ percentile being 72.1, 76.5, and 82.5 years. Of the sample, 523 (52.6%) were females, around 39.0% of participants had low education, and 43.1% participants lived alone. On average, each participant had two chronic diseases with the 25^th^, 50^th^, and 75^th^ percentile being one, two, and three chronic diseases. Out of the 0–5 range of the frailty phenotype, 35.7% of the participants presented without any frailty component and 31.3% of the participants had one criterion of frailty present. The median (IQR) for current frailty index was 0.2 (0.1 – 0.3) point out of the 0–1 range, grip strength was 24.0 (18.5 – 32.2) kg, walking speed was 9.0 (7.0 – 11.0) seconds, and MMSE was 28.0 (26.0 – 29.0) points out of the 0–30 range. Regarding three-year change, on average the frailty phenotype had worsened with the median (IQR) being 0.0 (0.0 – 1.0), the frailty index 0.03 (-0.01 – 0.07), grip strength weakened with -2.5 (-6.5 – 0.5), walking speed slowed 1.0 (0.0 – 3.0), and cognitive function as measured by MMSE 0.0 (-1.0 – 1.0). Supplementary Fig. S4 shows the distribution of current status and three-year change in health indicators stratified by sex: in general there were no significant differences between the sexes with the exception of current grip strength where males tended to be stronger than females.

**Table 1:** Characteristics of the study sample

**Characteristics**	
Total number of participants, n [%]	995 [100.0]
Age	76.5 (72.1; 82.5), 68.0 – 91.6
Age brackets	
– 1^st^ Tertile	70.6 (69.4; 72.0), 68.0 – 73.5
– 2^nd^ Tertile	76.5 (74.8; 78.4), 73.5 – 80.4
– 3^rd^ Tertile	84.5 (82.5; 87.2), 80.5 – 91.6
Female, n [%]	523 [52.6]
Education, n [%]	
– Low	388 [39.0]
– Medium	316 [31.8]
– High	291 [29.2]
Living alone, n [%]	417 [43.1]
Number of chronic diseases	2.0 (1.0; 3.0), 0.0 – 8.0
Health indicators – current status^a^	
Frailty Phenotype, n [%]	
– Score 0	355 [35.7]
– Score 1	311 [31.3]
– Score 2	179 [18.0]
– Score 3	104 [10.5]
– Score 4	43 [4.3]
– Score 5	3 [0.3]
Frailty Index (range 0 – 1)	0.2 (0.1; 0.3), 0.0 – 0.6
Grip strength, in kg	24.0 (18.5; 32.2), 1.0 – 62.5
Walking speed, in seconds	9.0 (7.0; 11.0), 3.0 – 102.0
Mini-Mental State Examination (range 0 – 30)	28.0 (26.0; 29.0), 9.0 – 30.0
Health indicators – three-year change^b^	
Frailty Phenotype	0.0 (0.0; 1.0), -3.0 – 4.0
Frailty Index	0.03 (-0.01; 0.07), -0.27 – 0.27
Grip strength	-2.5 (-6.5; 0.5), -28.0 – 11.5
Walking speed	1.0 (0.0; 3.0), -12.0 – 83.0
Mini-Mental State Examination	0.0 (-1.0; 1.0), -15.0 – 7.0

Data are presented as median (interquartile range), min – max, except as noted

^a^Current status of health indicators was measured in 1998

^b^Three-year change was the difference in health indicators  by subtracting the measurement in 1998 minus the measurement in 1995

**Fig. 1:**
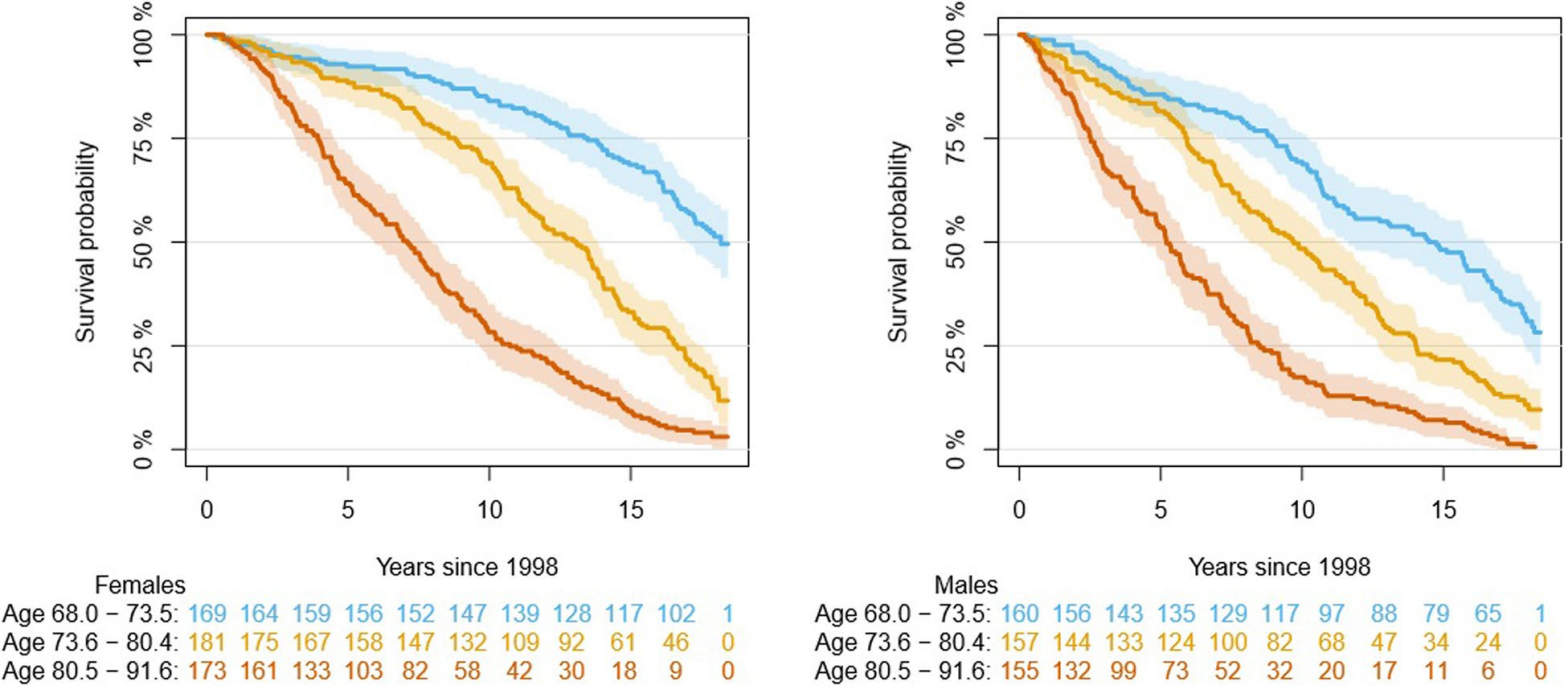
Kaplan Meier survival curves stratified by sex and age tertiles

**Fig. 2:**
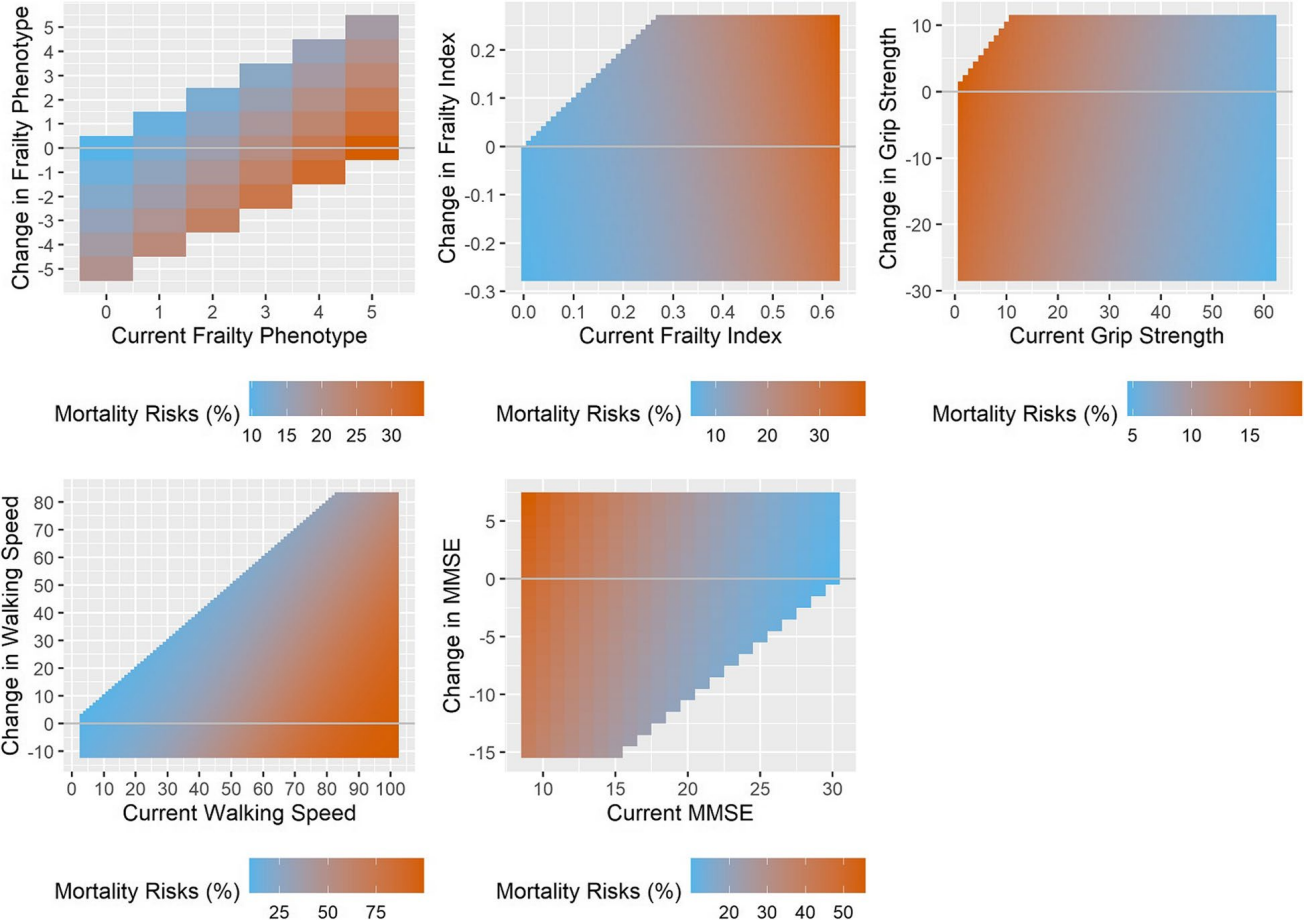
Five-year predicted mortality risks associated with current status and three-year change in all five health indicators for females aged 75 years old

Figure 1 shows Kaplan Meier survival curves stratified by sex and age tertiles. As expected, the oldest males had the lowest survival probability. Figure 2 shows five-year predicted mortality risks associated with current status and three-year changes in all health indicators for females aged 75 years old, given the majority of sex and average age of the population. This figure demonstrates that while three-year changes in health indicator scores (vertical axis) further worsened the predicted risks, it was not as big of an influence compared to the current status. Overall, the worse the current status (horizontal axis), the higher the predicted mortality risks. Regardless of a person’s three-year change, the mortality risks would be high primarily because of their current health status. This applies to the frailty phenotype, the frailty index, walking speed, and MMSE. As for grip strength, it appears that there was a threshold effect where if a person’s current grip strength was very low (below 10), then no matter how much the person had improved over the past three years, their mortality risks would still be high.

Table 2 shows the added value of current status and three-year change in health indicators to mortality prediction based on age and sex alone. Within five and ten years, 220 respectively 466 out of the 995 participants had died. Age and sex alone hold a discrimination ability of 72.8% AUC (95% CI 69.0 – 76.5) for five-year mortality and it was 77.7% AUC (74.8 – 80.5) for ten-year mortality. When predicting five-year mortality, the added AUC of current status of each of the several health indicators ranged from 0.7% for walking speed to 3.3% for the frailty index. This was consistently higher compared to the added AUC of three-year change in each of health indicators, ranging from -0.0% for the frailty phenotype to 1.1% for the frailty index. When the current status and three-year change were combined, their added AUC was similar to current status alone, from 0.6% for walking speed to 3.2% for frailty index and it also yielded the most significant p-values. Similar patterns were found for predicting ten-year mortality. The frailty index appears to improve mortality prediction the most, followed by the frailty phenotype, MMSE, grip strength, and walking speed. When adding all information on current status and three-year change of the various health indicators, AUC improved from 72.8 (69.0 – 76.5) to 77.6 (73.6 – 80.8) and 77.6 (74.8 – 80.5) to 80.5 (77.8 – 83.2) for five-year and ten-year mortality respectively.

**Table 2:** Added value of health indicators to mortality prediction based on age and sex

**Model**	**Health Indicator**	**Five-year Mortality** (220 died)		**Ten-year Mortality** (466 died)	
		**AUC**^**a**^** (95% CI)**	***P***-value^**b**^	**AUC**^**a**^** (95% CI)**	***P***-value^**b**^
Demographic variables	Age and sex	72.8 (69.0 – 76.5)		77.7 (74.8 – 80.5)	
+ Current status	+ Frailty Phenotype	+ 1.6 (0.6 – 2.6)	0.001	+ 0.8 (0.0 – 1.6)	0.050
	+ Frailty Index	+ 3.3 (1.6 – 4.9)	< 0.001	+ 1.8 (0.5 – 3.1)	0.005
	+ Grip strength	+ 1.0 (0.3 – 1.7)	0.005	+ 0.6 (0.0 – 1.2)	0.040
	+ Walking speed	+ 0.7 (-0.0 – 1.4)	0.060	+ 0.4 (-0.1 – 0.8)	0.100
	+ MMSE	+ 1.2 (0.2 – 2.3)	0.020	+ 1.1 (0.4 – 1.8)	0.002
+ Three-year change	+ Frailty Phenotype	- 0.0 (-0.2 – 0.1)	0.400	- 0.1 (-0.2 – 0.0)	0.200
	+ Frailty Index	+ 1.1 (0.1 – 2.1)	0.030	+ 0.5 (-0.3 – 1.3)	0.200
	+ Grip strength	+ 0.3 (-0.0 – 0.7)	0.090	+ 0.2 (-0.1 – 0.5)	0.100
	+ Walking speed	+ 0.4 (-0.1 – 0.9)	0.090	+ 0.1 (-0.2 – 0.4)	0.500
	+ MMSE	+ 0.1 (-0.3 – 0.6)	0.600	- 0.0 (-0.4 – 0.3)	0.900
+ Current status + Three-year change	+ Frailty Phenotype	+ 1.9 (0.6 – 3.1)	0.003	+ 0.9 (0.0 – 1.9)	0.050
	+ Frailty Index	+ 3.2 (1.6 – 4.8)	< 0.001	+ 1.7 (0.5 – 3.0)	0.008
	+ Grip strength	+ 0.9 (0.1 – 1.6)	0.020	+ 0.5 (-0.1 – 1.1)	0.100
	+ Walking speed	+ 0.6 (-0.2 – 1.3)	0.100	+ 0.4 (-0.2 – 0.9)	0.200
	+ MMSE	+ 1.2 (0.2 – 2.3)	0.020	+ 1.2 (0.5 – 1.9)	0.001

^a^*AUC* Discrimination ability as measured using Area Under the Curve in percentages

^b^*P*-values indicate the significance of the difference between a model containing age and sex plus a health indicator and the reference model that is age and sex only given the null hypothesis that the health indicator has no added discrimination ability

Table 3 divided the study population into age tertiles. Here the discrimination ability of residual age and sex alone in predicting five-year mortality was 65.2% AUC (56.8 – 73.5) in the youngest age tertile, 61.2% AUC (53.1 – 69.4) in the middle age tertile, and further decreased to 59.4% (53.3 – 65.5) in the oldest age tertile. The current status of the health indicators holds the highest added AUC in the oldest age tertile ranging from 2.3% for walking speed to 11.5% for the frailty index whereas this pattern was not as apparent in the younger age tertiles. With very few exceptions the added AUC of three-year changes in the various health indicators was lower when compared to the current status. Overall, the AUCs of demographic variables and health indicators combined did not exceed 71% in any of the different age tertiles. Similar patterns were found for ten-year mortality (data not shown). The order of health indicators in improving mortality predictions were the same as for the whole study population (Table 2), with the frailty index leading, followed by the frailty phenotype, MMSE, grip strength, and walking speed.

**Table 3:** Added value of health indicators to mortality prediction stratified by age tertiles

Strata	Health Indicator	Current Status		Three-year Change	
		**Average Score** **(min—max)**	**Five-year AUC**^a^	**Average Score** **(min—max)**	**Five-year AUC**^a^
Age tertile 68.0 – 73.5	Age and sex		65.2 (56.8 – 73.5)		65.2 (56.8 – 73.5)
(329 at risk, 36 died)	+ Frailty Phenotype	0.7 (0.0 – 4.0)	+ 0.2 (-1.8 – 2.2)	0.3 (-3.0 – 4.0)	- 0.8 (-2.0 – 0.3)
	+ Frailty Index	0.16 (0.00 – 0.61)	+ 2.0 (-4.4 – 8.5)	0.02 (-0.23 – 0.27)	- 0.3 (-2.9 – 2.4)
	+ Grip strength	28.6 (3.0 – 62.5)	+ 0.4 (-1.3 – 2.0)	-3.3 (-23.5 – 10.0)	+ 0.6 (-1.5 – 2.6)
	+ Walking speed	8.3 (4.0 – 48.0)	- 0.1 (-4.0 – 3.8)	1.3 (-8.0 – 37.0)	+ 1.5 (-1.2 – 4.2)
	+ Mini-Mental State Examination	27.9 (18.0 – 30.0)	- 1.4 (-6.3 – 3.5)	-0.0 (-9.0 – 6.0)	- 1.6 (-3.5 – 0.2)
Age tertile 73.6 – 80.4	Age and sex		61.2 (53.1 – 69.4)		61.2 (53.1 – 69.4)
(338 at risk, 50 died)	+ Frailty Phenotype	1.0 (0.0 – 4.0)	+ 0.7 (-3.2 – 4.6)	0.3 (-2.0 – 3.0)	- 0.6 (-2.4 – 1.1)
	+ Frailty Index	0.20 (0.02 – 0.61)	+ 3.8 (-0.7 – 8.2)	0.03 (-0.19 – 0.27)	+ 2.8 (-1.4 – 7.1)
	+ Grip strength	26.1 (1.0 – 49.5)	+ 1.6 (-1.6 – 4.9)	-3.1 (-28.0 – 11.5)	+ 0.1 (-2.7 – 3.0)
	+ Walking speed	9.5 (3.0 – 39.0)	- 0.7 (-3.1 – 1.6)	1.8 (-9.0 – 19.0)	- 0.3 (-1.3 – 0.6)
	+ Mini-Mental State Examination	27.2 (16.0 – 30.0)	+ 3.0 (-1.8 – 7.8)	-0.3 (-9.0 – 6.0)	+ 1.6 (-1.5 – 4.6)
Age tertile 80.5 – 91.6	Age and sex		59.4 (53.3 – 65.5)		59.4 (53.3 – 65.5)
(328 at risk, 134 died)	+ Frailty Phenotype	1.8 (0.0 – 5.0)	+ 7.9 (4.2 – 11.7)	0.5 (-2.0 – 4.0)	- 0.2 (-0.6 – 0.2)
	+ Frailty Index	0.26 (0.03 – 0.63)	+ 11.5 (6.5 – 16.5)	0.04 (-0.27 – 0.25)	+ 4.6 (1.4 – 7.8)
	+ Grip strength	20.9 (1.0 – 45.0)	+ 3.5 (0.5 – 6.5)	-4.4 (-21.0 – 6.5)	+ 0.0 (-0.8 – 0.8)
	+ Walking speed	13.1 (4.0 – 102.0)	+ 2.3 (0.1 – 4.4)	3.4 (-12.0 – 83.0)	+ 1.4 (-0.2 – 3.1)
	+ Mini-Mental State Examination	25.7 (9.0 – 30.0)	+ 3.9 (0.5 – 7.3)	-0.8 (-15.0 – 7.0)	- 0.4 (-1.7 – 0.9)

^a^*AUC* Discrimination ability as measured using Area Under the Curve in percentages

Supplementary analyses were performed. When the study population was stratified by sex, the added AUC of most health indicators was highest among males except for MMSE which was highest among females. There was no clear pattern when stratified by number of chronic diseases (see Supplementary Tables S5 and S6).

## Discussion

Among a representative population of community-dwelling older adults aged 68 years and older, we observed that changes in health indicators in previous years further increased mortality risks. However, it was not as big of an influence compared to the current health status. The AUC of age and sex alone for five-year mortality predictions was 72.8% AUC (95% CI 69.0 – 76.5) and the added AUC of the current status of health indicators ranged from AUC 0.7 (walking speed) to 3.3% (frailty index). The added AUC of three-year changes in health indicators was lower, ranging from AUC -0.0 (frailty phenotype) to 1.1% (frailty index), whereas the added AUC of both three-year change and current status combined was similar to current status alone, ranging from AUC 0.6 (walking speed) to 3.2% (frailty index). Across age groups, the added AUC of current status was highest in the oldest old, however there was no clear pattern with the three-year changes. When stratified by sex, the added AUC of most health indicators was highest among males except for MMSE which was highest among females. There was no clear pattern when stratified by number of chronic diseases. Findings for ten-year mortality were similar. Overall the frailty index appeared to improve mortality predictions the most, followed by the frailty phenotype, MMSE, grip strength, and walking speed.

### Predictive capabilities of demographic variables and health indicators

We found that the current health status measured at one time point performed better in accurately predicting mortality than change in health indicators over time, though the added predictive capabilities were limited. In the younger age group, age and sex captured most of the discrimination ability to predict mortality. In contrast, in the oldest old these demographic variables lose their predictive values while the health indicators gained predictive value. Since there is a severe lack of studies comparing predictive capabilities of demographic variables and health indicators separately [30], more research is needed to illuminate this finding. When age is driving the absolute mortality risks over the lifespan, it is important to carefully disentangle the predictive capabilities of health indicators from that of age. Taken together, the discrimination ability for the demographic variables and health indicators combined did not exceed AUC of 71% in any of the age groups. This means that at best, seven out of ten times the model correctly distinguishes the older individual who will die or survive in the next five years, indicating a modest ability to predict mortality [31].

### Comparison among functional indicators

We found that the frailty index improved mortality prediction the most, followed by the frailty phenotype, MMSE, grip strength, and walking speed. Both of these well-validated frailty indices were superior and the added values were highest in the oldest old. This is likely due to the all-encompassing nature of these indices designed to capture frailty; a disorder characterised by vulnerability to poor resolution of homeostasis after a stressor event as a consequence of cumulative age-related decline occurring pervasively in inter-related physiological systems [32]. Frailty indices are frequently perceived as interchangeable but each have different intended purposes and can actually be used to supplement each other [33]. The frailty index describes the presence of clinical conditions and characteristically acts as a marker of accumulated deficits and in line with this reasoning we found it to be the most discriminative in predicting mortality. The frailty phenotype is designed to specifically detect the risk of adverse outcomes in non-disabled individuals and is considered to be more appropriate for use when deciding the need of adapted care or interventions in clinical practice.

### Varying degrees of functional decline

In the current study, approximately half of the community-dwelling persons had varying degrees of frailty and also experienced varying degrees of functional decline in the following three-year period. Other studies have shed light on the differences in trajectories of functional decline and how it relates to underlying pathogenesis [34, 35]. For example, individuals with advanced frailty characteristically experience a slowly progressive functional decline with only a slight acceleration as death approaches. However, current prevention systems are typically targeted towards individuals with fast, severely declining functions. Therefore it is not suitable for many older adults at risk of dying due to progressive frailty [36]. More research is needed to observe these patterns of functional decline to develop prevention systems that takes into account these variations of functional decline and underlying pathogenesis.

### Implications for clinical practice

In this study, we found that the added discrimination of the various health indicators to mortality predictions based on age and sex alone tend to be limited. If a patient has to agree to go forward with certain medical interventions, the typical question would be “How likely is my chance to benefit from this intervention?”. In this case, a highly discriminating prediction model would best help the patient [37]. Even if health indicators would be able to substantially improve mortality predictions based on age and sex, there is still a laborious hurdle to overcome on whether these instruments actually help in clinical decision making. The key question is whether the use of models improves the outcomes of our interventions, particularly in older adults. Considering that the health indicators investigated in this study already fell short of expectations on its discrimination abilities, implementation in clinical practice should be regarded with caution.

### Strengths and limitations

We analyzed demographic variables, current functioning status, and changes over time separately as well as in combination, to explore their predictive abilities. Overall functioning indicated by the frailty indices, upper and lower body functioning measured by grip strength and walking speed, and cognitive functioning measured by MMSE were all compared to assess its performance in predicting mortality. In addition to that, all health indicators were well-operationalized, directly measured, and well-validated. Mortality data was complete, and therefore censoring information was available for everyone. Mortality differences between LASA participants and the general Dutch population never exceeded 1%, meaning the study is representative for the Dutch older population [38]. However, we used difference scores which have been known to lack reliability and this may have influenced results, especially for health indicators with very little change (e.g., MMSE). While it may provide more reliable information to assess trajectories of change in health indicators using more than two assessments during a longer period, we decided to study three-year change instead of six-year change or more since observing a three-year change is likely to be more compatible with clinical situations than monitoring change for longer period and it would also decrease the sample size to be even smaller thereby compromising power. The fact that we observed only modest changes in health indicators over a period of 3 years could be due to floor or ceiling effects, however, ceiling effects are not likely as only a small proportion of the study sample reported a very high number of health problems (e.g., only 0.3% of the sample had the highest score of 5 on the frailty phenotype). Finally, our study sample consisted of people who participated in both the 1995/1996 and the 1998/1999 interviews. Therefore, the sample does not include people who died between these two measurement waves, which could have led to underestimation of changes in health indicators.

## Conclusion

In conclusion, among community-dwelling persons aged 68 years and older, current health status improved mortality predictions based on age and sex better than three-year change and it was most prognostic in the oldest old. Although the added value of functional measures was highest in the oldest old, this added value was limited.

## Supplementary Information

**Additional file 1:** **Supplementary Figure S1.** Selection of Study Sample. **Supplementary Table S2.** Items Included inthe Frailty Index. **Supplementary Figure S3.** Study Design Scheme. **Supplementary Figure S4.** Distribution of Current Status and Three-year Change in Health Indicators Stratified for Sex. **Supplementary Table S5.** Added Value of HealthIndicators to Mortality Prediction Stratified for Sex. **Supplementary Table S6. **Added Value of Health Indicators to Mortality Prediction Based on Age and Sex Stratified for Number of Chronic Diseases.

## Data Availability

The datasets generated during the current study are not publicly available due to confidentiality, but the data underlying the results presented in this study are available from the Longitudinal Aging Study Amsterdam (LASA) and may be requested for research purposes. More information on data requests can be found on the LASA website: www.lasa-vu.nl.

## Abbreviations

AUC: Area under the curve
CI: Confidence interval
IQR: Interquartile range
LASA: Longitudinal Aging Study Amsterdam
MMSE: Mini-Mental State Examination
SD: Standard deviation
