# Supplementary material for: Do changes in frailty, physical functioning, and cognitive functioning predict mortality in old age? Results from the Longitudinal Aging Study Amsterdam
Source: BMC Geriatr. 2022 Mar 12;22:193. doi: 10.1186/s12877-022-02876-0 (PMC8917670; doi:10.1186/s12877-022-02876-0)
Supplement: Supplementary file 1 — Additional file 1: Supplementary Figure S1. Selection of Study Sample. Supplementary Table S2. Items Included inthe Frailty Index. Supplementary Figure S3. Study Design Scheme. Supplementary Figure S4. Distribution of Current Status and Three-year Change in Health Indicators Stratified for Sex. Supplementary Table S5. Added Value of HealthIndicators to Mortality Prediction Stratified for Sex. Supplementary Table S6. Added Value of Health Indicators to Mortality Prediction Based on Age and Sex Stratified for Number of Chronic Diseases. [file 12877_2022_2876_MOESM1_ESM.docx]

**Supplementary Figure S1. Selection of Study Sample**

**
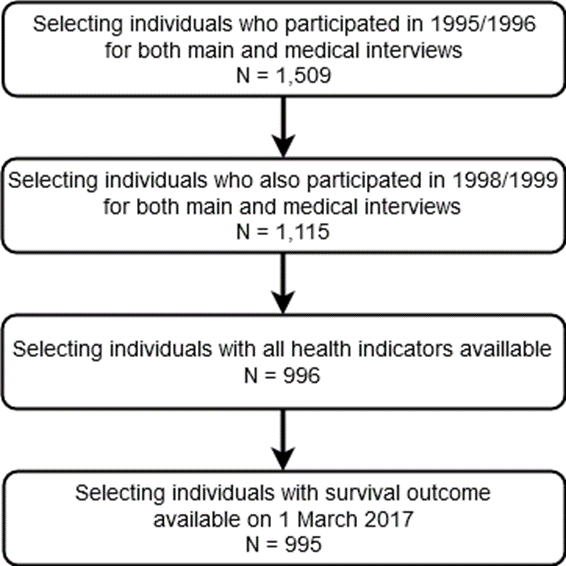
**

**Supplementary Table S2. Items Included in the Frailty Index**

| ADLs/IADLs | SELF-RATED HEALTH & SYMPTOMS |
| --- | --- |
| - Dressing | - Self-rated health |
| - Cut toenails | - Self-rated health compared to others |
| - Walking outside of the house | - Low physical activity |
| - Getting up from chair | - Slow gait speed |
| - Climb one flight of stairs | PSYCHOLOGICAL SYMPTOMS |
| - Use public transport | - Felt depressed |
| CHRONIC CONDITIONS | - Felt everything was effort |
| - Heart disease | - Happy (reverse-coded) |
| - Peripheral arterial disease | - Lonely |
| - High blood pressure | - Could not get going |
| - Stroke | - Enjoyed life (reverse-coded) |
| - Diabetes | COGNITION |
| - Chronic lung disease | - Delayed word recall |
| - Arthritis | - Self-rated memory |
| - Cancer | - Orientation regarding time |
| - Incontinence | - Orientation regarding place |
| - Other chronic disease 1 | - Attention |
| - Other chronic disease 2 | TOTAL NUMBER OF ITEMS: 32 |

Additional information on items and cut-points can be found here: Hoogendijk, E.O., Theou, O., Rockwood, K. et al. Development and validation of a frailty index in the Longitudinal Aging Study Amsterdam. Aging Clin Exp Res 2017;29;927–933. https://doi.org/10.1007/s40520-016-0689-0

**Supplementary Figure S3. Study Design Scheme**

**
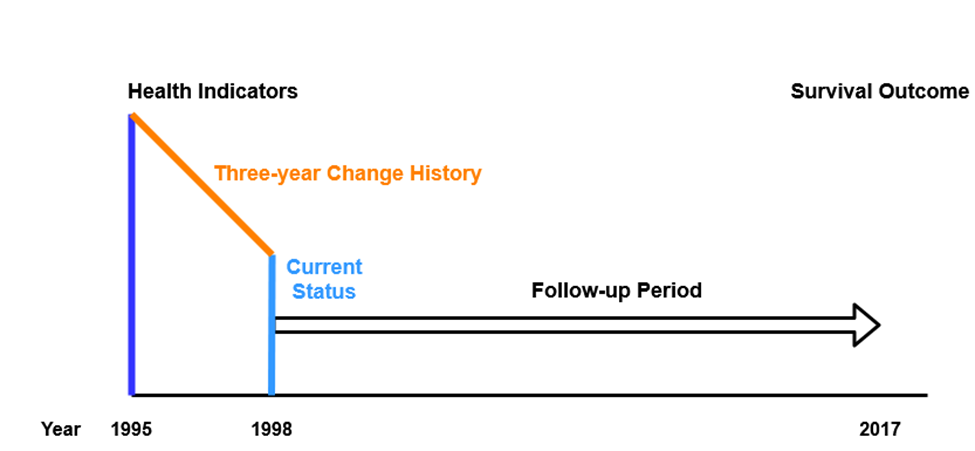
**

**Supplementary Figure S4. Distribution of Current Status and Three-year Change in Health Indicators Stratified for Sex**

**
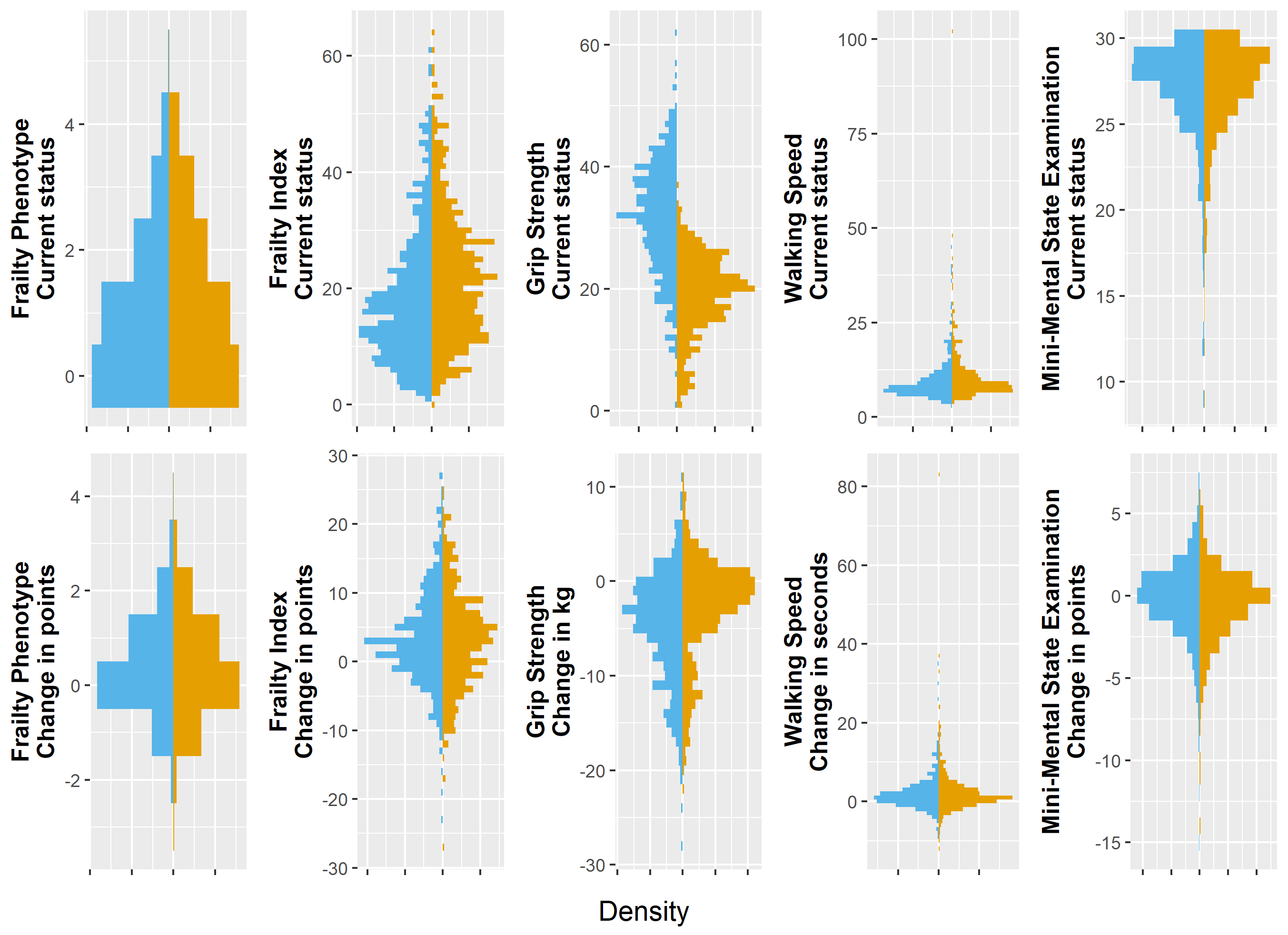
**

Males are displayed in blue and females are displayed in orange.

**Supplementary Table S5. Added Value of Health Indicators to Mortality Prediction Stratified for Sex**

| **Strata** | **Health Indicator** | **Current Status** | |  | **Three-year Change** | |
| --- | --- | --- | --- | --- | --- | --- |
|  |  | **Average Score**  **(min - max)** | **Five-year AUC**^1^ |  | **Average Score**  **(min - max)** | **Five-year AUC**^1^ |
| Males | Age |  | 71.3 (66.0 – 76.7) |  |  | 71.3 (66.0 – 76.7) |
| (472 at risk, 124 died) | + Frailty Phenotype | 1.1 (0.0 – 5.0) | + 2.7 (1.0 – 4.4) |  | 0.4 (-2.0 – 4.0) | + 0.2 (-0.2 – 0.7) |
|  | + Frailty Index | 0.18 (0.01 – 0.61) | + 4.4 (1.8 – 7.0) |  | 0.03 (-0.23 – 0.27) | + 2.6 (0.6 – 4.5) |
|  | + Grip strength | 32.0 (1.0 – 62.5) | + 1.2 (0.1 – 2.3) |  | -4.7 (-28.0 – 11.0) | + 0.8 (0.1 – 1.5) |
|  | + Walking speed | 9.5 (3.0 – 45.0) | + 1.5 (0.3 – 2.7) |  | 2.0 (-9.0 – 35.0) | + 1.2 (0.4 – 2.1) |
|  | + Mini-Mental State Examination | 27.0 (9.0 -30.0) | + 0.3 (-0.9 – 1.4) |  | -0.2 (-15.0 – 7.0) | + 0.0 (-0.5 – 0.6) |
|  |  |  |  |  |  |  |
| Females | Age |  | 73.7 (68.4 – 79.1) |  |  | 73.7 (68.4 – 79.1) |
| (523 at risk, 96 died) | + Frailty Phenotype | 1.2 (0.0 – 5.0) | + 0.8 (-0.4 – 1.9) |  | 0.4 (-3.0 – 4.0) | ̶ 0.2 (-0.4 – 0.0) |
|  | + Frailty Index | 0.22 (0.00 – 0.64) | + 2.3 (0.2 – 4.5) |  | 0.03 (-0.27 – 0.25) | ̶+ 0.2 (-0.7 – 1.0) |
|  | + Grip strength | 19.1 (1.0 – 37.0) | + 1.0 (0.0 – 2.0) |  | -2.6 (-21.5 – 11.5) | ̶ 0.1 (-0.6 – 0.4) |
|  | + Walking speed | 11.0 (4.0 – 102.0) | - 0.2 (-0.6 – 1.1) |  | 2.3 (-12.0 – 83.0) | + 0.0 (-0.4 – 0.5) |
|  | + Mini-Mental State Examination | 26.9 (9.0 – 30.0) | + 2.3 (0.4 – 4.2) |  | -0.5 (-14.0 – 6.0) | + 0.1 (-0.7 – 0.9) |

^1^ AUC: Discrimination ability as measured using Area Under the Curve in percentages

**Supplementary Table S6. Added Value of Health Indicators to Mortality Prediction Based on Age and Sex Stratified for Number of Chronic Diseases**

| **Strata** | **Health Indicator** | **Current Status** | |  | **Three-year Change** | |
| --- | --- | --- | --- | --- | --- | --- |
|  |  | **Average Score**  **(min - max)** | **Five-year AUC**^1^ |  | **Average Score**  **(min - max)** | **Five-year AUC**^1^ |
| With ≤ 1 Chronic Disease | Age and sex |  | 71.4 (64.1 – 78.6) |  |  | 71.4 (64.1 – 78.6) |
| (388 at risk, 59 died) | + Frailty Phenotype | 0.9 (0.0 – 5.0) | + 1.6 (-0.2 – 3.4) |  | 0.3 (-2.0 – 3.0) | ̶ 0.2 (-0.4 – 0.0) |
|  | + Frailty Index | 0.13 (0.00 – 0.57) | + 2.0 (-0.6 - 4.6) |  | 0.01 (-0.27 – 0.24) | + 0.1 (-0.4 – 0.5) |
|  | + Grip strength | 26.7 (1.0 – 55.5) | + 0.6 (-0.7 – 1.9) |  | -3.2 (-21.5 – 11.0) | + 0.1 (-0.6 – 0.9) |
|  | + Walking speed | 9.0 (4.0 – 40.0) | + 1.2 (-0.1 – 2.6) |  | 1.5 (-9.0 – 30.0) | + 0.4 (-0.1 – 1.0) |
|  | + Mini-Mental State Examination | 26.9 (9.0 – 30.0) | + 2.3 (-0.3 – 4.9) |  | -0.4 (-12.0 – 6.0) | + 0.6 (-0.6 – 1.9) |
|  |  |  |  |  |  |  |
| With 2 Chronic Diseases | Age and sex |  | 75.2 (68.8 – 81.6) |  |  | 75.2 (68.8 – 81.6) |
| (298 at risk, 67 died) | + Frailty Phenotype | 1.1 (0.0 – 5.0) | + 0.2 (-0.7 – 1.0) |  | 0.4 (-3.0 – 4.0) | ̶ 0.3 (-0.6 – 0.0) |
|  | + Frailty Index | 0.20 (0.06 – 0.51) | + 1.2 (-0.5 – 2.9) |  | 0.04 (-0.19 – 0.27) | + 1.5 (-0.3 – 3.3) |
|  | + Grip strength | 25.4 (1.0 – 62.5) | + 0.0 (-0.4 – 0.5) |  | -3.8 (-23.5 – 10.0) | + 0.0 (-0.6 – 0.6) |
|  | + Walking speed | 10.6 (3.0 – 102.0) | + 0.1 (-0.9 – 1.1) |  | 2.5 (-8.0 – 83.0) | ̶ 0.1 (-0.8 – 0.6) |
|  | + Mini-Mental State Examination | 27.1 (12.0 – 30.0) | + 2.0 (0.3 – 3.8) |  | -0.3 (-15.0 – 6.0) | + 0.0 (-1.4 – 1.4) |
|  |  |  |  |  |  |  |
| With > 2 Chronic Diseases | Age and sex |  | 70.0 (63.6 – 76.3) |  |  | 70.0 (63.6 – 76.3) |
| (309 at risk, 94 died) | + Frailty Phenotype | 1.6 (0.0 – 4.0) | + 1.6 (-0.3 – 3.5) |  | 0.5 (-3.0 – 3.0) | - 0.3 (-0.6 – 0.1) |
|  | + Frailty Index | 0.30 (0.11 – 0.64) | + 2.6 (-0.1 – 5.2) |  | 0.06 (-0.17 – 0.27) | + 0.3 (-1.2 – 1.8) |
|  | + Grip strength | 23.2 (1.0 – 57.5) | + 2.0 (-0.0 – 4.0) |  | -3.9 (-28.0 – 11.5) | + 0.4 (-0.3 – 1.1) |
|  | + Walking speed | 11.7 (4.0 – 48.0) | + 0.1 (-0.9 – 1.2) |  | 2.6 (-12.0 – 37.0) | + 0.3 (-0.6 – 1.1) |
|  | + Mini-Mental State Examination | 26.8 (9.0 – 30.0) | + 0.2 (-1.2 - 1.6) |  | -0.4 (-14.0 – 7.0) | - 0.1 (-0.3 – 0.1) |

^1^ AUC: Discrimination ability as measured using Area Under the Curve in percentages
